# Successful Treatment of Sarcoma by Electrolysis and Cataphoresis Combined with the Internal Use of Donovan’s Solution*Read before the American Surgical Association, May 6, 1897.

**Published:** 1897-09

**Authors:** J. McFadden Gaston

**Affiliations:** Atlanta, Ga.; Professor of Principles and Practice of Surgery in the Southern Medical College


### Southern Medical Record.

A MONTHLY JOURNAL OF MEDICINE AND SURGERY.

VOL. XXVII. ATLANTA, GA., SEPT., 1897. No. 9

### Original Articles.

### SUCCESSFUL TREATMENT OF SARCOMA BY ELECTROLYSIS AND CATAPHORESIS, COMBINED WITH THE INTERNAL USE OF DONOVAN'S SOLUTION.*

BY J. McFADDEN GASTON, M. D., ATLANTA, GA.

Professor of Principles and Practice of Surgery in the Southern Medical College.

The distinguishing features of benign and malignant tumors are supposed to depend upon the difference in origin, and while other abnormal growths result from the development of micro-organisms they have no part in these neoplasms.

The various benign tumors are produced from tissues corresponding to those of their seat, and are styled homologous, while the malignant tumors have different elements from the surrounding structures, being designated as heterologous formations.

It is held by some pathologists that the source of malignant growth is found in the fœtus, and that the pre-existing state leads ultimately to the modification of structure presented in postnatal conditions. Yet this does not seem compatible with the usual late appearance of malignant neoplasms.

Professor Senn thinks that the definition of a tumor should explain its origin, its histological characteristics, and its behavior towards its immediate environment. He refers to the different attempts to define tumors, and gives his own definition as follows: "A tumor is a localized increase of tissue, the

*Read before the American Surgical Association, May 6, 1897.

product of tissue-proliferation of embryonic cells of congenital or postnatal origin, produced independently of microbic causes."

This may convey a definite idea to those who are satisfied with Cohnheim's theory, but I must confess that it does not give me a clear conception of the essential pathological features of tumors, and I am disposed to hold on to the old, simple definition, which I have given for many years in opening my lectures upon tumors,—viz., "A tumor is an abnormal development of tissue, independent of the ordinary results of inflammation." This is free from ambiguity and contains no contested points, which can not be said of the above definition.

The treatment of uterine fibroids by the Apostoli methods of applying electricity has been attended with fairly good results, and the cauterizing effects of the electro-cautery has been generally satisfactory in the lesser cutaneous growths, but, so far as known to me, the field of malignant tumors has only recently been explored by electricity, so as to yield practical results.

Dr. G. Betton Massey directed attention to a new treatment of sarcoma, at the meeting of the Medical Society of Pennsylvania, May 21, 1896.[1] This is based upon the curative action of electricity, which "requires a soluble zinc electrode as positive pole, freshly coated with mercury at each application, and properly insulated. This electrode, of a size and shape adapted to each case, is inserted into the tumor after a small opening is made by negative electro-puncture, under the chloride-of-ethyl spray, and with a properly adjusted large negative pole, as indifferent pole, a current of from 100 to 200 milliamperes may be gradually turned on," and repeated daily, until a cure is effected.

"Under this method the tumor is gradually disintegrated, and much of it discharged through the opening; the area of induration about it shrinks progressively until finally the malignant growth is replaced by a healthy wound, which is ultimately allowed to heal, but not until the whole of the morbid tissue is destroyed or transformed into granulation tissue."

It is notable that the process adopted by Dr. Massey contemplates a comparatively rapid breaking down of the morbid structure and a disintegration with discharge of the *debris*, whereas the course pursued in a different class of cases by Dr. Robert Newman is only "to use weak currents of from three to four milliamperes, *seances* lasting not more than from five to ten minutes, with intervals of about one week or longer." He says that "electrolysis acts as a chemical decomposition by absorption, which never burns or destroys tissues. It is not followed by hemorrhage or other unpleasant consequences."

The internal use of the preparations of arsenic, urged especially by Lawson, and the employment hypodermically of the antitoxine by Coley, have been attended with benefit in some cases and failure in others.

The feasibility of curing a malignant growth without surgical interference by the knife or cauterization has been thought questionable; and yet there are some instances of the disappearance of tumors which were recognized as malignant neoplasms under the influence of constitutional and local measures which are worthy of consideration.

In view of facts indicating the practicability of treating malignant inoperable tumors by electrolysis and cataphoresis, I undertook the management of a case in which the diagnosis of sarcoma was previously made by two colleagues based upon macroscopic and microscopic tests.

A white boy of twelve years of age suffered for some months from a growth in the hypogastric region, and was placed under the care of Dr. J. B. S. Holmes, of this city. An exploratory incision was made by him diagonally across the right flank above the internal abdominal ring, and into the indurated mass of the tumor. It was found that it presented the characteristics of sarcoma, with such adhesions to the surrounding structures as to preclude any attempt to remove it by the knife. The incision was accordingly closed, and an unfavorable prognosis of the case was given to the parents of the boy. Dr. William Perrin Nicolson also examined him and fully concurred in the views of Dr. Holmes. It was afterwards determined to take the patient to Richmond, Va., where Dr. Hunter McGuire examined the case, and reopened the incision so as to procure a specimen of the tumor for microscopic investigation. He was impressed with the serious nature of the case, and declined to undertake any radical operation. The result of a careful examination of the specimen with the microscope by Dr. M. D. Hoge, Jr., of Richmond, indicated that the tumor was a small, round-celled sarcoma, and thus confirmed the previous diagnosis in the case.

This patient was turned over to me for examination on the 16th of November, 1895, and I found an abdominal tumor, extending from near the anterior inferior spinous process of the ilium and the adjacent portion of the pubic bone across to nearly the corresponding line on the opposite side, and reaching from near the symphysis pubis to the umbilicus, with marked induration of the mass. There was no mobility in the tumor, and adhesions seemed to exist between its anterior surface and the abdominal wall. An exact measurement of the outlines of in the the tumor gave in the transverse diameter six inches; longitudinal diameter, seven inches; in circumference, twenty-one inches.

The anterior surface was smooth and somewhat prominent, but without any salient projection. Moderate pressure by palpation was borne without flinching, and there was no complaint of sharp pain or discomfort in that region. The general condition of the boy was that of cachexia with pallor and weakness, but without any marked emaciation of body or limbs. With these facts before me, the history of the family, given by the father of the boy, threw no light upon the case, and I felt constrained to accept the view of the case taken by my colleagues, as reported by me.

It was evidently impracticable to do any radical surgical operation, and although I was not aware that Dr. Massey had then reported his cases, [2] nor had I received any report of success in the use of electricity for sarcoma, I came to the conclusion that it offered some prospect of relief in this case. Upon stating my conviction to the father, who is a very intelligent man, he manifested a favorable disposition towards accepting my proposition as a *dernier ressort*. My suggestion was adopted also in regard to combining an alternative course of internal treatment, with the resort to electrolysis locally, and the following prescription, which I have found to exert great resolvent influence in other cases, was ordered for the patient:

℞ Syrup trifolium compound. fluid ounces, 3½;
Donovan's solution (liq. ars. et hyd. iod.) fluid ounce ½.
SIG.—Mix, and take a small teaspoonful with water three times a day.

It may be stated that each fluidounce of the trifolium syrup contains the following: Red clover-blossoms, 32 grains; stillingia, 16 grains; lappa, 16 grains; phytolacca root, 16 grains; berberis aquifolium, 16 grains; casca amarga, 16 grains, xanthoxylum, 4 grains; iodide of potassium, 8 grains.

This was employed simply as a menstruum for the more active preparation of the double iodide of mercury and arsenic, known as liquor arsenici et hydrargyri iodidi in the United States Pharmacopœia. The patient got about one grain of iodide of potassium with eight drops of Donovan's solution in a teaspoonful of the mixture three times a day, and took it, without any disturbance of his stomach or bowels, continuously while electrolysis was applied to the tumor.

I am giving a somewhat minute account of the medical treatment in this case so as to enable others to judge of the influence of medication upon the result when considered in connection with the application of electricity locally. On the 17th of November, 1895, I commenced the use of electricity with a twelve-cell battery, manufactured by Waite and Bartlett, having the cups of glass, in which the zinc and carbon plates are immersed in a solution of bichromate of potassium, which was kindly furnished by Dr. J. A. Childs, of this city.

The needle representing the positive pole was introduced into the substance of the right side of the tumor, and the sponge electrode representing the negative pole, about two inches in diameter, was placed on the left margin of the tumor, having the connection of six cells, and keeping up the current for five minutes daily. *Seance* was gradually increased during the first week, and afterwards, until the time was extended to ten minutes; the punctures were made about half an inch apart, going around the outer border of the tumor; and after the 25th of November the applications were only made on every alternate day, until the 20th of April, 1896, when they were made every third day. In the meantime the needle was changed to the negative pole, and on December 29, 1895, Donovan's solution was commenced upon the positive pole, by saturating a small piece of cotton with it, and laying it upon the central portion of the moistened sponge electrode. This process is known as cataphoresis, by which medicinal substances are introduced into the body with the galvanic current. Knowing that Dr. Hunter McGuire had obtained satisfactory results in the treatment of goitre by this means, I wrote to him for information as to the method of procedure, and he kindly furnished me with an account of the process adopted in his cases, which has served as a guide in my application of the medicated electricity in this case.

Having no means of estimating the voltage or amperage. with the resistance by ohms, I depended upon the manifestations of local effects, and graduated the number of cells accordingly. When the fluid has become weaker by repetition of the currrent the number of cells has been increased, and after renewal of the fluid fewer cells have again been employed in making the applications. Without regard, therefore, to an accurate scientific record the clinical result has been closely watched, and in the course of the treatment a larger battery has been used, so as to get the effect of a weaker current diffused over more space by having a larger number of cells included in the connection of the poles. This was resorted to when the force of the galvanic current was materially lessened by repeated application of the battery with the same fluid. And as many as thirty cells have thus been brought into the circuit without any marked inconvenience to the patient. The effect was marked by minute vesication alternated with small pits over the part to which the Donovan solution was applied, while the usual liberation of gas and some exudation of fluid occurred around the needle of the negative pole. After encircling the entire tumor with the punctures the needle was dispensed with, and another sponge electrode substituted for it; when no sign of any irritation was apparent at the negative pole, but the vesication continued to appear at the seat of the positive electrode, when it was applied for as much as ten minutes, with the pledget of cotton, soaked in Donovan's solution, placed on the moist sponge.

During the application of the galvanic current with the needle, whether on the side of the positive or negative pole, there was pain to a marked degree, and this was indicated by yells and screams on the part of the boy from the beginning to the end of the *seance* so as to attract the attention of all the neighbors. There was less suffering with the use of the positive and negative sponge electrodes, and eventually the twelve-cell battery was changed for my own of thirty cells, with a gradual increase to fifteen, twenty, and thirty cells in the circuit, applied every five days. The internal administration of the syrup of trifolium compound and Donovan's solution was, in the month of February, 1896, changed for a combination of the same quantity of Donovan's solution with five and a half ounces of succus alterans, which consists of the following in each pint: Sixteen troy ounces of stillingia sylvatica, smilax sarsaparilla, phytolacca decandra, lappa minor, and xanthoxylum Carolinianum (Eli Lilly). A teaspoonful of this mixture was taken three times a day. It will be observed that the chief difference of this from the other compound is that the iodide of potassium is omitted. I observed, in studying authorities upon this matter, that Dalton included among the incompatibles with Donovan's solution the salts of the alkalies, and hence I used as a menstruum the succus alterans.

In the mean time notable changes were observed in the size and density of the tumor, and in the general condition of the patient. The border line had receded decidedly from its original limits, excepting at its encroachments upon the horizontal ramus of the right pubic bone, where it remained in such close proximity as to give the impression to the touch that the tumor was attached to the bone.

The measurements, on March 1, 1896, of the tumor, made by my son, Dr. J. McF. Gaston, Jr., gave for the longitudinal diameter five inches; for the transverse diameter, four and a half inches; for circumference, fifteen and a half inches; showing the great reduction in the respective dimensions of two inches, one and a half inches, and five and a half inches from those of November 18, 1895, being three and a half months.

Another measurement, made the 29th of July, 1896, by my son, was as follows: Longitudinal, median line, three and a half inches; transverse line, at broadest line, three inches; circumference, ten and a half inches; making a reduction, in four months, in the respective dimensions, of one and one-half inches, one and one-half inches, and five inches, in the previous measurement. The mobility of the small mass remaining was now very notable when the tumor was caught up between the thumb and fingers; but there was still an attachment between the tumor and the parietal wall, and some slight oozing of a serous nature from the outer end of the incision. This appeared to discharge from the substance of the tumor, and indicates persistent degeneration in it.

The general health of the patient has very materially improved, and having undergone examination at different periods since being under my care by Drs. Hunter McGuire, J. B. S. Holmes, Arch and J. C. Avary, they all coincided in the favorable progress of the case.

Upon furnishing the details of my treatment in this case to Dr. G. Betton Massey he expressed his gratification at my success, and regards it as a confirmation of the claims made in his paper,* to which I have referred, and also in a previous one read before the Philadelphia County Medical Society, January 9, 1895. He infers correctly that I had not seen the earlier paper, thus affording another instance of the independent conception of another novelty in surgery. This point has been illustrated by my previous experience on several occasions, but my view is none the less original from being anticipated by another, with also priority of publication.

My case illustrates another phase of electrolysis and cataphoresis. There was a marked diminution in the induration of the tissue and in the general prominence of the mass, with less discharge from the incision which had been made by my colleague before the case was placed under my care, and had only partially closed by granulation.

It should be noted that the only restriction made in the diet of the patient from the outset of the treatment was to exclude all greasy food and highly seasoned dishes, and the digestion was good during the use of the medicine and cataphoresis for more than a year without interruption. He has only taken a teaspoonful of the succus alterans and Donovan's solution once a day for the past three months, and has used the medicated electricity by cataphoresis once a week, for fifteen minutes at each *seance*. During the month of March he was laid up with an attack of mumps (parotitis) for more than a week and suspended the regular treatment for the appropriate remedies to combat this acute condition, but the use of this special course has since been resumed, and he has returned to his duties at school, presenting no traces of disease of a local or constitutional nature at the present time, May 1, 1897.

We are prepared by the foregoing considerations to make the following inferences:

(1) That the diagnosis of sarcoma being made by clinical observation and confirmed by the microscope, there ought not to exist any question as to the nature of the disease.

(2) The evident adhesion of the tumor to the abdominal viscera, and especially to the front wall of the abdomen, pre-

---

*Dr. Massey presented a paper at the meeting of the American Medical Association, May, 1897, in further demonstration of the result of his mode of treatment.

cluded surgical interference with the knife, and thus placed the case under the class of inoperable tumors.

(3) Having a reasonable ground to expect some benefits in malignant growths from medicinal treatment, the combination of mineral and vegetable alteratives affords the best prospect of relief in this condition.

(4) The history of the treatment of benign tumors with the continuous galvanic current gives encouragement for its application in cases of a malignant type, and especially in that variety of neoplasm known as sarcoma, in which its efficacy has been recently tested.

(5) It is not requisite to employ a destructive or disintegrating effect of the continuous electric current to secure the favorable action upon tumors, and the process of electrolysis may be initiated with a few cells and gradually increased without disintegration at either electrode.

(6) This treatment may be commenced with the needle at the positive pole and the sponge electrode at the negative pole of the Waite & Bartlett battery, and so placed as to pass the current through the substance of the tumor. After the daily application thus for a week or ten days, the needle may be used with the negative pole and the sponge with the positive pole, making the applications on alternate days.

(7) The employment of cataphoresis, in which medication is applied upon the positive sponge electrode, while either the needle or sponge electrode is connected with the negative pole, induces some irritation of the skin when the medicine is applied and conveys its properties into the substance of the tumor.

(8) While the exact estimate of the force of the electric current would promote scientific results, the clinical observation of the effects upon the tissues subserves all practical purposes in the treatment of tumors by electrolysis and cataphoresis; and it is not requisite to be provided with the appliances for measuring the voltage and amperage or ohms of resistance in the use of the battery. The time occupied in accomplishing a cure by the moderate current of electricity is greater than that which would be taken in a more violent application, yet the freedom from any destructive process renders the former more satisfactory to the patient.

(9) My case, added to those already reported in which medicated electricity has been efficient in the removal of malignant tumors, should warrant the profession in resorting to this mode of treatment; I can further vouch for the efficacy of internal medication with electrolysis and cataphoresis in the treatment of sarcoma.

(10) Theoretic views in regard to the origin of malignant tumors should not prove a barrier to the adoption of general and local measures of treatment, which have been employed with satisfactory results in a case clearly diagnosticated to be sarcoma. Clinical observations in the progressive development of the neoplasm, with macroscopic and microscopic tests, clearly indicate its malignant character.

(11) The process of atrophic decomposition and reabsorption of the sarcomatous structures under the operation of the medicated electric current favor the expectation that tumors of a carcinomatous nature may undergo like changes with similar treatment. We are, therefore, warranted in resorting to cataphoresis as a tentative measure of treatment for all kinds of malignant neoplasms.

REFERENCES.

1. American Medico-Surgical Bulletin, June 27, 1896.

2. Medical News, March 9, 1895; Journal of the American Medical Association, August 24, 1895; Philadelphia Polyclinic, October 19, 1895.

